# Baden-Baden

**Published:** 1894-08-11

**Authors:** 


### Continental Notes from a Holiday Correspondent.

### BADEN-BADEN.

Baden-Baden lies in the little valley of the Oos, between two spurs of the Black Forest, and is one of the most beautifully placed towns in Europe. Though its hot mineral springs have been known since Roman times, it is only since the abolition of the gambling saloons in 1872 that it has attained great popularity as a health resort, but its popularity is now second only to that of Wiesbaden, and it is visited by about fifty or sixty thousand persons annually. The season is May to October, but we found it by no means crowded in June. There are a large number of springs, of temperatures varying from 110 to 150 deg. Fahr., and giving a total output of nearly a quarter of a million gallons daily. In character the water is alkaline and saline, the chief constituent being chloride of sodium, of which there are 20 to 30 parts in 10,000 parts of water. There is also a high percentage of arsenic and lithium. The water is drunk to some extent, but the principal use to which it is put is for hot baths. Besides the Friedrichs Baths—the finest bath house in Europe—there are small bath houses in connection with all the chief hotels. These baths are principally used for gout and rheumatism of every form, and undoubtedly are often of great benefit. It is open to question, however, whether the benefit is not due more to the heat of the water than to any virtue in its mineral constituents, and there can be no question whatever that the beneficial effect of the baths is largely augmented by the good air of the Black Forest, and the regular exercise, plain food, and early hours rigorously prescribed for all bathers.

The place of greatest interest to medical visitors to Baden-Baden is the above-mentioned Friedrichsbad, which is a model bath-house. It stands on the site of the old Roman baths on the side of the hill from which the principal springs issue. The supply of water is practically boundless, and arrangements are accordingly made for its free use. The right and left wings of the building, which are exactly similar, are devoted to women and men respectively. On the first floor are ordinary plunge baths—large marble basins sunk in the floor, with a constant current of hot water flowing through them. There are also sitz baths and electric baths, and an inhaling room. On the second floor are large baths for several bathers, swimming baths, Turkish, mud, Russian, and every other recognized form of bath. In all these the temperature of the water can be regulated exactly, as may be ordered by the physician. The swimming baths are fixed at four temperatures—59, 80, 95, and 100 deg. Fahr., and the steam baths at 112 to 126 deg. Fahr. On the second floor is also placed the Swedish gymnasium, arranged, like the baths, in two sections, for men and women. This gymnasium was fitted up in 1884 with apparatus brought from Stockholm, a most fearful and wonderful assortment of machinery for active and passive exercise and mechanical massage. For instance, one may see a rheumatic old gentleman put on a saddle, and bobbed up and down by steam power, or at a later stage in his "cure" he may have to work himself up and down with treadles as on a tricycle. Or, again, if massage to any special part is desired, he may be fixed tightly in a seat and the part rolled, rubbed, or beaten with little indiarubber-covered rollers or hammers worked at a high speed by steam. This last procedure seems rather barbarous, but as a matter of fact is not at all unpleasant, as the hammers are so well padded and the pressure so light. One very curious machine is designed to stir up the circulation in the abdominal organs. Everyone knows the appearance of a mortar-mill, where a large upright wheel rolls round and round in a sort of huge saucer of mortar. The machine I allude to is on this principle, the wheel being of wood, covered with rubber, and about ten inches in diameter, while instead of a saucer, it works over the abdomen of the patient, who reclines lightly clad on his back on a couch! On each machine is a sand-glass timed to run three minutes, this being the time allotted to each exercise.

No doubt one may say that a good walk, or row, or a game of tennis in the open air would be as good, but every physician knows that prescriptions of that sort are seldom carried out unless the patient is so inclined, while here the exercise is so exact, and can be so carefully suited to any one limb or joint, that it is easy to get patients to undertake it.

We came away from the Friedrichsbad greatly pleased with all we had seen, and wondering that so few English are found among the visitors.

Baden-Baden has the reputation of being dear, but by inquiry beforehand one can easily secure pension at moderate prices, either at an hotel or at one of the numerous villa boarding houses. For a few days' stay, when it is not worth while making pension arrangements, Cook's hotel coupons will protect one from overcharges, and the hotel for which they are available is most comfortable.

The walks round the town are endless and most charming. The chief ones are marked with lines of coloured paint on the trees in connexion with Professor Oertel's "Terrain Cure" for lung and heart diseases. A series of yellow marks shows that the path is a level one, red marks that it is an easy ascent, or yellow and red together that it is a steep climb. By the aid of these marks and a map of the environs, walks can be chosen to suit any person, healthy or invalid.

